# Synthesis and Characterization of the Inclusion Complex of Dicationic Ionic Liquid and β-Cyclodextrin

**DOI:** 10.3390/ijms11103675

**Published:** 2010-09-28

**Authors:** Puvaneswary Subramaniam, Sharifah Mohamad, Yatimah Alias

**Affiliations:** University of Malaya Centre for Ionic Liquids, Department of Chemistry, Faculty of Science, University of Malaya, 50603 Kuala Lumpur, Malaysia; E-Mails: sharifahm@um.edu.my (S.M.); yatimah70@um.edu.my (Y.A.)

**Keywords:** inclusion complex, β-cyclodextrin, dicationic ionic liquid, host-guest system

## Abstract

The supramolecular structure of the inclusion complex of β-cyclodextrin (β-CD) with 1,1′,2,2′-tetramethyl-3,3′-(*p*-phenylenedimethylene) diimidazolium dibromide (TetraPhimBr), a dicationic ionic liquid, has been investigated. The inclusion complex with 1:1 molar ratio was prepared by a kneading method. Fourier transform infrared spectroscopy (FTIR), powder X-ray diffraction (XRD) analysis, ^1^H NMR and thermogravimetric analysis (TGA) confirmed the formation of the inclusion complex. The results showed that the host-guest system is a fine crystalline powder. The decomposition temperature of the inclusion complex is lower than that of its parent molecules, TetraPhimBr and β-CD individually.

## 1. Introduction

**Figure 1:**
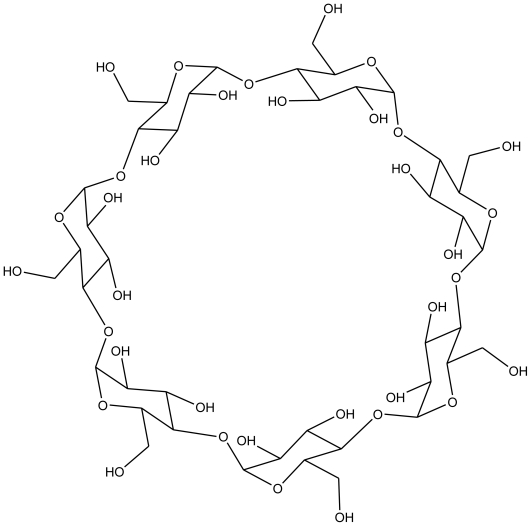
The structure of β-cyclodextrin (β-CD).

Inclusion complexes (ICs), particularly those leading to supramolecular self-assemblies, have been attracting much attention as they serve as models for understanding molecular recognition and as precursors for designing novel nanomaterials for electronics and biological applications [1–4]. An example of supramolecular self-assembly is host-guest inclusion complexes made of cyclodextrins (CDs) and guest molecules. CDs are macrocyclic oligosaccharides composed of 6, 7 or 8 D(+)-glucose units linked by α-1,4-linkages and named α-, β- or γ- CD, respectively. The internal cavity of CD is hydrophobic and can accommodate various organic and inorganic guest molecules, while the upper and lower rims are formed by the secondary and primary OH groups and therefore are hydrophilic (Figure 1) [5]. Recently, with an increasing interest in understanding the mechanism of molecular recognition, complexes of CDs have been investigated extensively in fields such as pharmaceutical formulations for drug delivery, cosmetics, catalysis, food manufacturing, enzyme simulation and organic synthesis [5–7].

Ionic liquids (ILs), which consist of organic cations and appropriate anions, have increased interest due to their high ionic conductivity, excellent chemical stability, nonflammability and negligible volatility [8,9]. As they promise significant environmental benefits, ILs have been developed as a green and recyclable alternative to traditional organic solvents in various applications ranging from synthesis, electrochemistry, catalysis, materials, separations and biotechnology, to the nuclear industry [10,11].

To date, many investigations have been conducted into the inclusion complexation and encapsulation interaction in cyclodextrin chemistry [4]. There are only a few studies that have previously reported on the inclusion interaction between CDs and ILs [12,13], though there have been some reports on the combination of CDs and ILs [14–17]. However, inclusion complexation of dicationic ionic liquids by β-cyclodextrin has not been reported yet. Multifunctional ionic liquids, especially dicationic and dianionic ionic liquids, have been shown to have a greater range of physical properties than most traditional, singly charged ionic liquids [18,19]. They often have greater thermal stability, lower volatility, and more flexibility in tuning/varying their physicochemical properties. Considering the special structure and properties of ILs, it is of interest to investigate the complexation behavior between a dicationic IL and CD.

**Figure 2:**
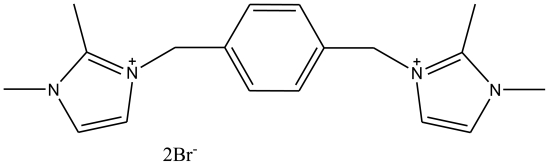
The structure of 1,1′,2,2′-tetramethyl-3,3′-(p-phenylenedimethylene) diimidazolium dibromide (TetraPhimBr).

In this work, we present the supramolecular encapsulation of 1,1′,2,2′-tetramethyl-3,3′-(*p-*phenylenedimethylene) diimidazolium dibromide (TetraPhimBr)(Figure 2) by β-cyclodextrin (β-CD). The formation of the inclusion complex of β-CD-TetraPhimBr was confirmed by various physico-chemical techniques including fourier transform infrared spectroscopy (FTIR), powder X-ray diffraction (XRD), ^1^H NMR spectroscopy and thermogravimetric analysis (TGA).

## 2. Results and Discussion

### 2.1. FTIR Analysis

**Figure 3:**
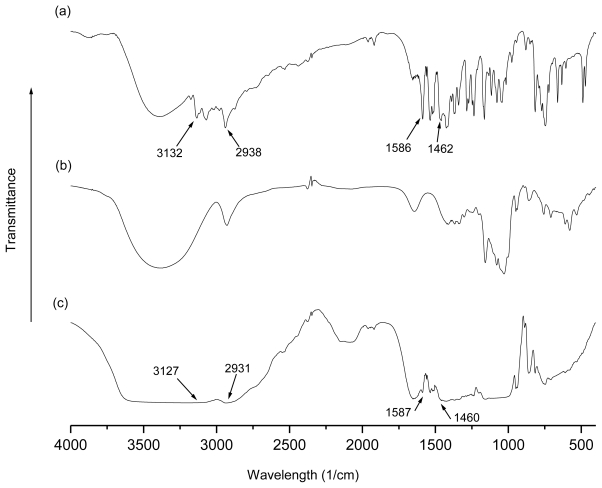
FTIR spectra of (**a**) TetraPhimBr, (**b**) pure β-CD and (**c**) β-CD-TetraPhimBr.

FTIR is a useful technique used to confirm the formation of inclusion complex [20]. Figure 3 shows the FTIR spectra of β-CD, TetraPhimBr and β-CD-TetraPhimBr. The FTIR spectrum of β-CD-TetraPhimBr is similar to that of pure β-CD, which is a major characteristic for the host-guest inclusion complex of CDs. The presence of TetraPhimBr in the inclusion complex is further confirmed when the most obvious bands of CH imidazolium ring stretch, CH_3_(N) stretch, imidazolium ring bend and CH_2_ bend of TetraPhimBr are observed in the FTIR spectrum of the β-CD-TetraPhimBr inclusion complex at 3127, 2931, 1587, 1460 cm^−1^, respectively. These bands are shifted upon inclusion complexation compared to the bands in the free ionic liquid [13]. The broader O-H stretching band of the inclusion complex in the range of 3000–3600 cm^−1^ corresponds to the multiple O-H functional groups of β-CD molecules [21]. Furthermore, the FTIR curves in the fingerprint region (below 1300 cm^−1^) confirm that the β-CD-TetraPhimBr is different from the originating parent molecules, as they possess different spectroscopic signals [22].

### 2.2. XRD Analysis

**Figure 4:**
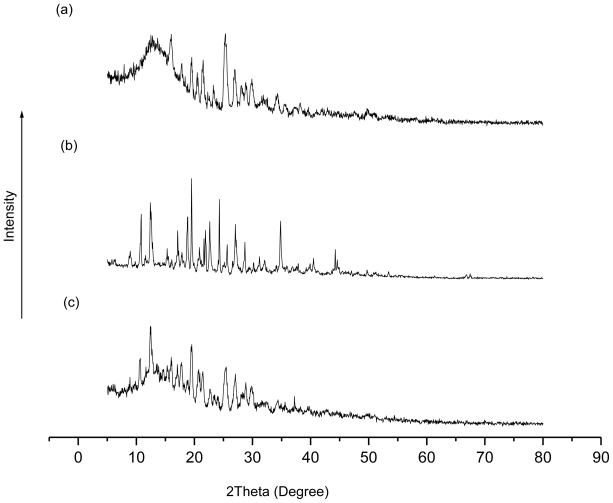
X-ray diffractograms of (**a**) TetraPhimBr, (**b**) β-CD and (**c**) β-CD-TetraPhimBr.

Further evidence of complex formation was obtained by X-ray powder diffraction [22]. The crystal structures of CD complexes are classified mainly into three types: channel-type, cage-type and layer-type. Figure 4 shows the XRD patterns of β-CD, TetraPhimBr and β-CD-TetraPhimBr. The results suggest that the inclusion complex obtained is a fine crystalline powder and the XRD pattern of the β-CD-TetraPhimBr shows that the inclusion complex forms head-to-head channels [23]. The XRD pattern of the β-CD-TetraPhimBr inclusion complex is different from the XRD patterns of its parent molecules and this confirms the formation of IC and that the β-CD-TetraPhimBr inclusion complex crystallizes with a structure that is different from its components [22,24].

### 2.3. Solid-State ^13^C CP/MAS (Cross-Polarization Magic-Angle Spinning) NMR Spectroscopy

**Figure 5:**
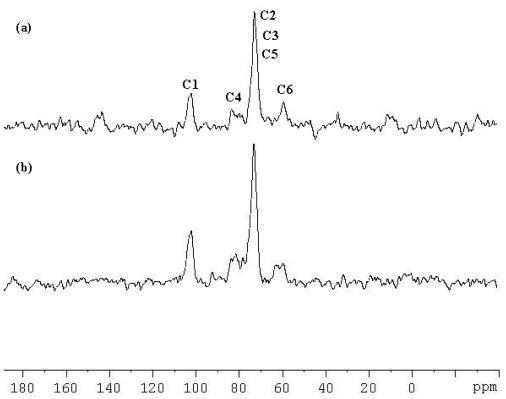
^13^C CP/MAS NMR spectra of (**a**) β-CD-TetraPhimBr and (**b**) β-CD.

^13^C CP/MAS NMR spectroscopy gives information about complexation of CDs [12,23,25]. Figure 5 shows the solid-state ^13^C CP/MAS NMR spectra of uncomplexed β-CD and the inclusion complex of β-CD-TetraPhimBr. The β-CD molecule is known to assume a less symmetrical conformation in the uncomplexed state. The splitting for all C1–C6 resonances in the spectrum of β-CD reflects resolved carbon resonances from each of the glucose units. In contrast, the resolved resonances disappear in the spectrum of β-CD-TetraPhimBr and each carbon of the glucose unit is observed as a single peak. The results indicate that the β-CD molecules adopt a more symmetrical conformation upon complexation, and each glucose unit of β-CD is in a similar environment, which further supports the formation of inclusion complex between β-CD and TetraPhimBr [12,23,25].

### 2.4. ^1^H NMR Spectroscopy

**Figure 6:**
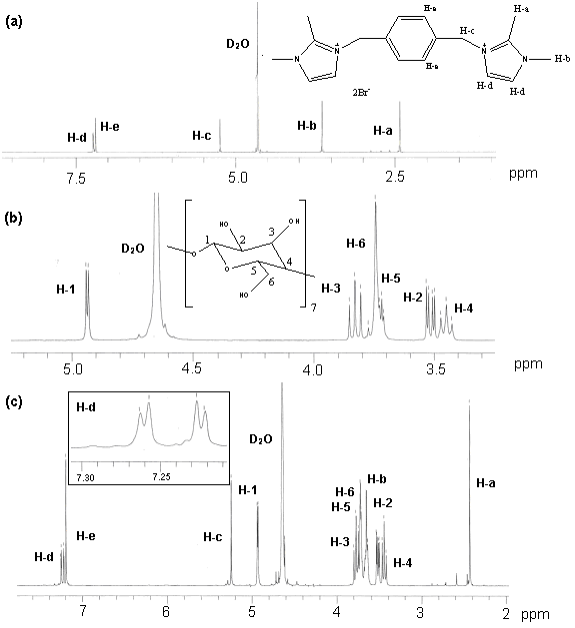
400-MHz ^1^H NMR spectra of **(a)** TetraPhimBr, **(b)** β-CD and **(c)** β-CD-TetraPhimBr in D_2_O.

Figure 6 shows the ^1^H NMR spectra of β-CD, TetraPhimBr and β-CD-TetraPhimBr in D_2_O. In the ^1^H NMR spectrum of the β-CD-TetraPhimBr inclusion complex, the presence of hydrogen atom signals belonging to both β-CD and TetraPhimBr molecules strongly confirmed that the inclusion complex has formed. Comparison of these spectra reveals that all proton signals in TetraPhimBr shifted upfield (Table 1) in the presence of β-CD.

The upfield shift of the protons lying on the inner surface of β-CD, *i.e.*, H-3, H-5 and H-6, results from shielding effects, suggesting that TetraPhimBr is included into the cavity of β-CD. The H-2 and H-4 protons of β-CD are located outside the cavity, thus their signals remain unchanged upon addition of guest molecules [26].

In Figure 6(a), the doublet of proton signals of the imidazolium ring of TetraPhimBr appeared as a singlet-like signal because of the fast rotation rate of the imidazolium ring relative to the NMR time scale. It is considered that the rotation rate of the imidazolium ring is slowed by the β-CD-TetraPhimBr inclusion complex formation, and as a result, those of imidazolium ring signals appear as a normal NMR pattern of the IL moiety in Figure 6(c).

The broadening of the proton signals of IL was not observed, indicating that in β-CD-TetraPhimBr inclusion complex the motions of the protons of TetraPhimBr are not restricted and thus it maybe fits loosely with β-CD [26].

In addition, the ratio of TetraPhimBr to β-CD in inclusion complex was determined by their integration which confirms the inclusion complex of ratio 1:1 [25].

### 2.5. TG Analysis

**Figure 7:**
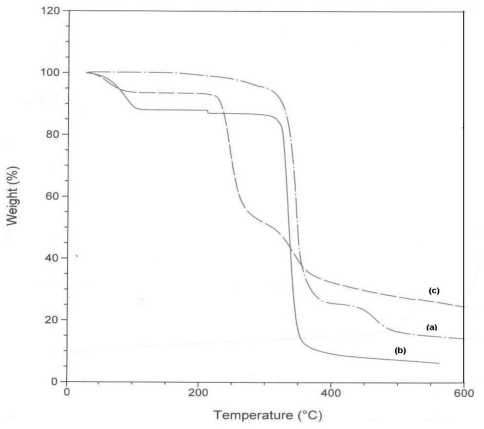
TGA curves of **(a)** TetraPhimBr, **(b)** β-CD and **(c)** β-CD-TetraPhimBr.

The thermal stability of β-CD-TetraPhimBr was evaluated by TGA and the results were compared with pure β-CD and free TetraPhimBr. Figure 7 shows the weight-loss curves for β-CD-TetraPhimBr and its precursors. It is quite interesting that β-CD-TetraPhimBr has a lower initial decomposition temperature than both pure β-CD and free TetraPhimBr. Normally the initial decomposition temperatures of inclusion complexes are higher than those of CD, and the inclusion complexation is believed to contribute to the better stability of CDs [27,28]. Thus, the present TGA results are unusual. This phenomenon may results from the unique molecular structure and properties of the ionic liquid compared to the organic compounds and polymers [29]. The steric congestion and the geometry distortion of the components may make β-CD-TetraPhimBr unstable. Moreover, the detachment of ions pairs of TetraPhimBr upon inclusion complexation may reduce the thermal stability of β-CD-TetraPhimBr. Similar phenomenon has been observed in the study of inclusion complex of β-CD with ionic liquid [12].

## 3. Experimental Section

### 3.1. Materials

β-Cyclodextrin (99%) was purchased from Acros (Hungary). Other reagents and chemicals were of analytical reagent grade and were used as received.

### 3.2. Measurements

The IR spectra were recorded on a Perkin–Elmer RX1 FT-IR spectrometer with samples prepared as KBr pellets. All the spectra were run in the range of 400–4000 cm^−1^ at room temperature. The ^1^H NMR spectra were recorded in D_2_O on a Lambda JEOL 400 MHz FT-NMR spectrometer. XRD patterns were taken using Cu Kα irradiation with a Siemens D5000 X-ray diffractometer (voltage, 40 kV; current, 100 mA). Powder samples were mounted on a sample holder and scanned from 5° to 80° at a speed of 3° per minute. ^13^C CP/MAS NMR spectra were acquired on a Bruker AV-400 NMR spectrometer with a sample spinning rate of 8.0 kHz at room temperature. Thermogravimetric analyses (TGA) were made using a TA Instruments Q500. Samples were heated at 20 °C/min from room temperature to 600 °C in a dynamic nitrogen atmosphere.

### 3.3. Methods

#### 3.3.1. Preparation of the TetraPhimBr Ionic Liquid

TetraPhimBr was prepared as reported previously. α,α-Dibromo-*p*-xylene and 1,2-dimethylimidazole (1:2.1) were refluxed in DMF (50 mL) for 3 h. The reaction product which precipitated as a white powder was filtered, washed with diethyl ether and dried under vacuum. Crystals of TetraPhimBr were grown from water [30]. Yield: 75%

#### 3.3.2. Preparation of the β-CD-TetraPhimBr Inclusion Complex

The inclusion complex of TetraPhimBr with β-CD was prepared by the kneading method [31]. β-CD was wetted with ethanol in agate mortar and kneaded to form a paste. Then equimolar of TetraPhimBr and ethanol were added. The sample was kneaded for approximately 30 min and dried at 105 °C.

## 4. Conclusions

An inclusion complex was formed between the TetraPhimBr ionic liquid and β-CD with the ratio of 1:1. Fourier transform infrared spectroscopy (FTIR), powder X-ray diffraction (XRD) analysis, ^1^H NMR spectroscopy and thermogravimetric analysis (TGA) confirmed the presence of a new product, with properties different from those of originating host and guest molecules. The results obtained by different characterization techniques clearly indicate that the kneading method leads to the formation of complex between TetraPhimBr ionic liquid and β-CD. XRD results show that the host-guest system is a fine crystalline powder. XRD and ^13^C CP/MAS NMR indicated that the inclusion complex possessed a channel-type structure and β-CD molecule in the inclusion complex adopted a symmetrical conformation, each glucose unit of β-CD is in a similar environment. TGA studies illustrate that the thermal stabilities of the inclusion complex is lower than its two precursors.

## Figures and Tables

**Table 1:** The chemical shifts (δ) of β-cyclodextrin, TetraPhimBr and β-CD-TetraPhimBr.

	β-cyclodextrin	TetraPhimBr	β-CD-TetraPhimBr	
	
	δ	δ	δ	Δδ
H-1	4.94		4.94	0
H-2	3.52		3.52	0
H-3	3.83		3.79	−0.04
H-4	3.45		3.45	0
H-5	3.72		3.69	−0.03
H-6	3.75		3.73	−0.02

H-a		2.45	2.43	−0.02
H-b		3.66	3.62	−0.04
H-c		5.25	5.23	−0.02
H-d		7.25	7.24	−0.01
H-e		7.19	7.18	−0.01
